# Artificial Intelligence in Healthcare: From Predictive Models to Generative, Multimodal, and Agentic AI

**DOI:** 10.3390/bioengineering13101175

**Published:** 2026-10-08

**Authors:** İsmail Baydili, Burak Tasci, Gülay Tasci, Sengul Dogan, Turker Tuncer

**Affiliations:** 1 Department of Audiovisual Techniques and Media Production, Vocational School of Technical Sciences, Fırat University, Elazığ 23119, Türkiye; ibaydili@firat.edu.tr; 2 Vocational School of Technical Sciences, Fırat University, Elazığ 23119, Türkiye; 3 Department of Psychiatry, Elazığ Fethi Sekin City Hospital, Elazığ 23280, Türkiye; 4 Department of Digital Forensics Engineering, Technology Faculty, Fırat University, Elazığ 23119, Türkiye; sdogan@firat.edu.tr (S.D.); turkertuncer@firat.edu.tr (T.T.)

**Keywords:** artificial intelligence, medical diagnosis, differential diagnosis, prognostic stratification, generative AI, multimodal AI, agentic AI, clinical translation

## Abstract

Artificial intelligence (AI) is increasingly used across the diagnostic pathway, from screening and disease detection to differential diagnosis, prognostic stratification, and treatment-response assessment. This narrative review examines predictive, generative, multi-modal, and agentic AI through a diagnostic-science lens and evaluates the evidence required for translation into clinical practice. Predictive models can identify complex patterns in images, physiological signals, laboratory data, and electronic health records, but high retrospective accuracy does not establish diagnostic utility. Clinical validity and utility depend on the intended use, target population, disease spectrum and prevalence, reference standard, operating threshold, calibration, and the consequences of false-positive and false-negative results. Generative AI may support problem representation, differential diagnosis, information synthesis, and documentation, yet fluent outputs can omit critical alternatives, amplify false premises, or convey unjustified certainty. Multimodal systems may better reflect clinical reasoning by integrating imaging, text, signals, pathology, and molecular data, but they must demonstrate incremental value over the best single-modality test and remain robust to missing data. Agentic systems can coordinate evidence retrieval, test selection, and sequential workflows, thereby increasing the need for bounded permissions, auditability, error recovery, and human escalation. Across all paradigms, a credible pathway to diagnostic implementation requires independent external and prospective validation, representative consecutive patients, subgroup analysis, workflow and human–AI evaluation, clinical impact studies, and post-deployment surveillance. The field should therefore be judged not by model capability alone, but by whether AI measurably improves diagnostic yield, timeliness, safety, equity, and patient-relevant outcomes in real care settings.

## 1. Introduction

Artificial intelligence (AI) is increasingly used across biomedical research and clinical care, including diagnosis, prognosis, risk stratification, medical imaging, physiological signal analysis, and decision support [1,2,3,4]. Much of this progress has been driven by task-specific machine-learning and deep-learning models developed for well-defined clinical problems. In some areas, particularly image-based diagnosis, these systems have achieved performance comparable to that of healthcare professionals [5]. However, strong benchmark results do not always translate into clinical benefit, and more complex models are not necessarily better than simpler, well-designed approaches [6]. The focus has therefore shifted from accuracy alone to whether AI systems remain reliable across patients, institutions, and clinical settings and whether their outputs can meaningfully support patient care [2,4].

From a diagnostic science perspective, AI should be evaluated as part of a test-to-decision pathway rather than as an isolated classifier. Relevant use cases include population or opportunistic screening, triage, disease detection, differential diagnosis among competing etiologies, diagnostic confirmation, and prognostic or treatment-response stratification after diagnosis. Each use case has distinct requirements for the intended population, index test and reference standard, disease spectrum and prevalence, operating threshold, uncertainty, and the clinical consequences of false-negative and false-positive results [5,7,8,9,10]. A credible translation pathway must therefore connect the model output to a defined user, clinical decision, downstream action, and measurable patient or workflow outcome.

The emergence of foundation models and generative AI has substantially broadened this landscape. Large language models (LLMs) can perform multiple language-intensive tasks within a shared pretrained architecture, including clinical summarization, question answering, documentation, information synthesis, and decision support [11,12,13,14]. Their flexibility contrasts with the narrower functional scope of many conventional predictive systems, but also introduces distinct limitations. Fluent outputs may contain unsupported or incorrect information, vary with prompts and context, and provide limited insight into the provenance or calibration of generated responses [11,13,15]. In parallel, multimodal AI is extending model inputs beyond a single data type by integrating combinations of text, medical images, electronic health records, physiological measurements, pathology, and molecular information [16]. More recently, agentic AI has introduced the prospect of systems that can combine reasoning with planning, memory, retrieval, tool use, and sequential action [17,18,19]. These developments are not simply successive generations of the same technology. Rather, they represent increasingly interconnected capabilities—prediction, generation, multimodal integration, and goal-directed action—that may coexist within the same clinical AI ecosystem.

The various forms of predictive, generative, multimodal and agentic AI are rapidly converging, so that it is increasingly less useful to consider each in isolation. The growing array of forms of AI in health care is in itself not a problem. Rather, the familiar problems of clinical translation persist: performance may decrease in application to new populations, in new institutions, with new data; seemingly strong results may be attenuated by poor calibration, by bias, by data leakage, by a lack of external validation, or by poor fit to clinical work flows [5,6]. The relevant question is therefore not only whether an AI system performs well, but whether it remains reliable, useful, and safe when introduced into actual care.

Although all clinical AI systems are susceptible to error, the consequences become particularly important when model outputs directly influence high-stakes recommendations or clinical actions. Evaluation should therefore consider not only model capability but also the intended clinical context and the potential consequences of failure. Randomized studies of machine-learning-based decision support systems have shown their potential to influence clinically relevant processes and thus must be evaluated within the respective care processes rather than on the basis of retrospective criteria [20]. Reporting frameworks such as CONSORT-AI and SPIRIT-AI provide standards for trials and trial protocols involving AI interventions [21,22], while FUTURE-AI extends this perspective across the AI lifecycle, emphasizing fairness, universality, traceability, usability, robustness, and explainability [23]. For multimodal and agentic systems, these considerations increasingly apply to the whole clinical system, including interactions between data, models, external tools, clinicians, and patients.

This state-of-the-science narrative synthesis integrates current evidence across predictive, generative, multimodal, and agentic AI and provides a clinically oriented framework for evaluating their translation into healthcare practice. We assess how they contribute to disease detection, differential diagnosis, prognostic stratification, and treatment-response assessment, while also considering documentation, workflow, and governance functions that determine whether diagnostic information can be used safely. The paradigms are treated as convergent capabilities rather than successive generations. Across them, we distinguish technical performance from diagnostic validity, diagnostic utility, and clinical impact, and organize the translational pathway around intended use, credible reference standards, independent external and prospective validation, human–AI interaction, workflow integration, patient-relevant outcomes, and post-deployment surveillance. The central question is not whether increasingly sophisticated systems can generate a prediction or recommendation, but whether their use improves diagnostic decisions and patient care reliably, safely, and equitably.

The scope extends beyond diagnostic model performance to developments that may alter how AI is incorporated into healthcare practice. Accordingly, treatment planning, digital twins, embodied and robotic systems, and autonomous scientific discovery are considered where they represent a meaningful extension from model-level inference toward patient-specific, interactive, or increasingly autonomous forms of clinical and biomedical decision support. Their inclusion is intended to examine the expanding translational boundary of healthcare AI rather than to provide a comprehensive review of each emerging field. This distinction also allows established clinical applications and more exploratory technologies to be discussed within the same framework without implying equivalent levels of evidence or clinical maturity.

This review is structured to move from conceptual evolution to clinical translation. We first establish the diagnostic and translational framework used to evaluate healthcare AI, then examine predictive, generative, multimodal, and agentic AI according to their distinct capabilities, evidence base, and failure modes. Subsequent sections consider applications across clinical practice, cross-cutting requirements for trustworthy implementation, and emerging directions that may extend current models of AI-enabled care.

### Literature Search Strategy

A targeted literature search was conducted in Web of Science Core Collection, Scopus, and PubMed to identify relevant studies for each of the review themes. A set of appropriate search terms was defined for each section of the review. Where possible, primary studies providing clinically relevant data were given preference, particularly those reporting external validation, multi-center studies, prospective studies, randomized trials and implementation studies in clinical settings. Additionally, methodological papers and reviews from authoritative sources were included to address certain clinical areas where there is currently a lack of relevant clinical evidence and to provide context to the review of healthcare AI development and clinical application. Studies were selected on the basis of clinical relevance, methodological quality and relevance to the development and clinical application of healthcare AI. The literature was then narratively and integratively reviewed as opposed to a systematic review or meta-analysis. To strengthen the diagnostic focus, search terms and study selection also addressed screening and disease detection, differential diagnosis, diagnostic accuracy, reference standards, prognostic stratification, external and prospective validation, diagnostic workflow, and clinical implementation.

When multiple studies addressed similar questions, greater interpretive weight was given to evidence from independent external validation, multicenter studies, prospective evaluations, randomized or comparative studies, human–AI and workflow evaluations, and clinical implementation studies. Retrospective and proof-of-concept studies were included where they provided important evidence in emerging areas but were interpreted according to their corresponding level of clinical maturity. This evidence-prioritization approach was used to support a clinically oriented synthesis while maintaining the narrative and integrative nature of the review.

## 2. Evolution of Artificial Intelligence in Healthcare

Artificial intelligence in healthcare has evolved from relatively simple predictive models based on structured clinical data to more complex learning systems capable of processing large and heterogeneous healthcare datasets. Representation learning methods that enable direct extraction of relevant information from high-dimensional data such as medical images and signal data have been particularly successful. Furthermore, generative, multimodal, and agentic AI models have recently progressed to stages of content generation, multi-data fusion, and goal-directed actions, thereby significantly changing not only the scope of applications of AI in healthcare but also its clinical utilization and assessment. In diagnostic science, this evolution expands the claim under evaluation from single-endpoint classification to context-dependent differential diagnosis, multimodal evidence integration, and multi-step test-to-action pathways; accordingly, validation must remain anchored to the intended diagnostic use rather than to architectural novelty.

### 2.1. From Task-Specific Prediction to Representation Learning

The early expansion of artificial intelligence in medicine was dominated by task-specific models developed for a predefined input, endpoint, and clinical context. Conventional machine-learning pipelines typically depended on variables selected or engineered before model fitting, making performance closely coupled to the quality of data representation and the assumptions embedded in feature construction. This paradigm proved effective for structured clinical data and well-defined prediction problems, but became increasingly restrictive as biomedical datasets expanded in scale, dimensionality, and complexity [2,3,24].

Deep learning altered this relationship between data representation and prediction. Rather than relying primarily on manually specified features, multilayer neural networks enabled hierarchical representations to be learned directly from raw or minimally processed data. This shift was particularly consequential for information-rich modalities such as medical images and physiological signals, in which clinically relevant patterns are often distributed across high-dimensional inputs. Reviews of physiological-signal applications, for example, documented the increasing use of deep architectures across electrocardiographic, electroencephalographic, electromyographic, and electro-oculographic data and highlighted their capacity to exploit larger and more heterogeneous datasets. More broadly, the transition from conventional machine learning to deep learning has been characterized as a data-driven shift in which model development moved progressively from explicit feature design towards learned representations.

This transition did not eliminate the limitations of task-specific modelling. Most deep-learning systems remained optimized for a particular dataset and endpoint, and gains in representation learning did not inherently confer portability across institutions, populations, or tasks. As deployment experience accumulated, attention therefore moved from model architecture alone towards the data conditions under which models are developed and maintained. Zhang and colleagues framed this change as a shift from model-centric development towards a more data-centric perspective, emphasizing data availability, distribution shift, and the infrastructure required to sustain performance after deployment. Representation learning thus expanded the range of tractable medical data, but the prevailing paradigm remained largely one model for one clinical objective.

### 2.2. Transformers and Foundation Models as an İnflection Point

Transformers marked a more fundamental change because they enabled representations learned at scale to be reused across a wider range of downstream tasks. Initially developed for natural-language processing, transformer architectures were subsequently adapted to clinical text, electronic health records, medical imaging, physiological signals, and biomolecular sequences. A recent review of transformer-based healthcare applications documents this expansion across multiple forms of biomedical data and across tasks including diagnosis, reconstruction, report generation, and outcome prediction. Their importance for healthcare therefore extends beyond improvements in language modelling; transformers provided a scalable architecture for learning context-dependent representations from large and heterogeneous datasets.

Foundation models extended this principle further. Instead of training a model de novo for every clinical endpoint, large-scale pretraining creates a reusable representational substrate that can subsequently be adapted through fine-tuning, prompting, in-context learning, retrieval, or other task-specific mechanisms. This changes the unit of development from an isolated predictive model to a general pretrained model capable of supporting multiple downstream functions. In healthcare, this approach has been most visible in large language models, but the same principle increasingly applies to imaging, pathology, molecular data, and multimodal systems [11,15,25,26].

This distinction is crucial. In essence, the high parameter count of a foundation model does not automatically qualify every large neural network to be considered a foundation model. Rather, the key property of a foundation model is the transferability of the learned representations to other tasks, as well as (potentially) to other data domains. While the ability to learn to complete a large set of tasks in a single training run can potentially reduce the need to train many individual models to complete a single task, the sources of uncertainty in the model’s behavior are re-distributed. That is, in addition to the uncertainty inherent in any machine learning model, there is also uncertainty related to the pretraining data, the particular adaptation approach used for a given task, the model–task alignment, and how well the pretraining representations generalize to the specific clinical context in which they are applied. Thus, while the broad capability of a foundation or large language model to complete a wide variety of tasks is an important attribute, such broad capability does not automatically translate to clinical competence in all contexts [11,15,26,27]. Harrer, for example, argues that these systems are more appropriately developed as assistive technologies under human oversight than as substitutes for clinical decision makers.

### 2.3. From General-Purpose Models to İntegrated AI Systems

Once the performance of single tasks is improved by their corresponding AI models, the potential of various tasks is further increased by combining them. Generative models extended this capability by producing text, images, and structured outputs. Then, multimodal models can combine different data sources, while tool-enabled systems connect AI models with external knowledge, software and computing power. The various earlier AI approaches are not replaced but rather combined in clinical AI systems in order to increase their scope of application.

Multimodal AI systems aim to capture relationships between different sources of information that a clinician would normally interpret together. This could be, for example, medical images, narrative reports, structured health records, and molecular or physiological measurements over time. The type of information included would depend on the specific clinical problem being addressed [28]. Foundation-model approaches are increasingly being developed to align such information within shared or interoperable representational spaces, allowing a single system to support tasks that previously required separate modelling pipelines.

A further extension occurs when models are embedded within systems that maintain state, select tools, decompose tasks, and execute multi-step workflows. Biomedical AI-agent frameworks already describe systems in which language and generative models are combined with structured memory, scientific knowledge, computational tools, and experimental platforms. This architecture changes what constitutes an AI system: the clinically relevant object may no longer be one model producing one output, but an orchestrated sequence of models, data sources, tools, and human interventions. Recent healthcare literature similarly distinguishes single-turn conversational systems from modular, multistep agents capable of tool use and bounded workflow execution.

**Figure 1:**
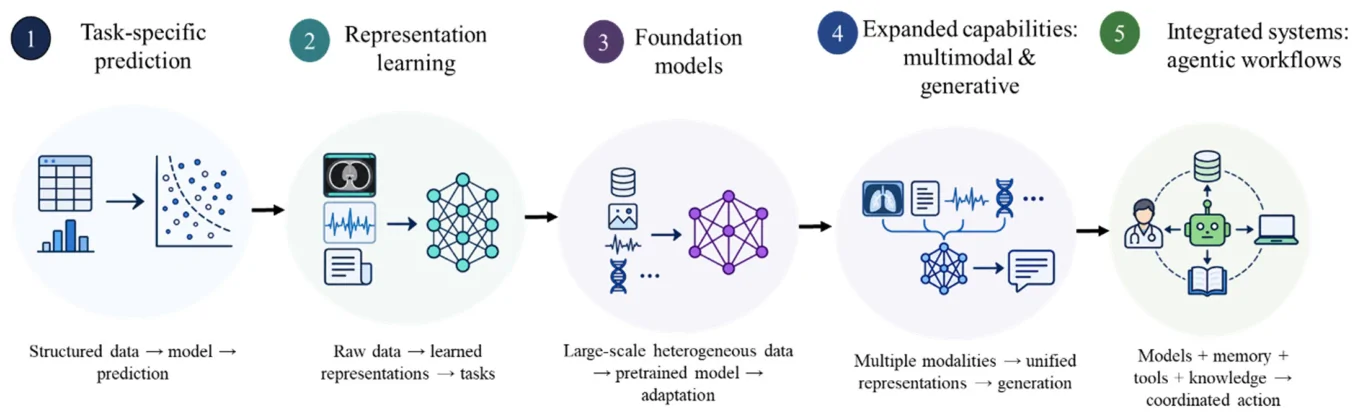
Evolution of artificial intelligence capabilities in healthcare.

The more consequential change is the widening scope of what an AI system can coordinate. Healthcare AI has progressed from learning a mapping between inputs and outcomes towards building reusable representations and, increasingly, systems that can combine generation, heterogeneous evidence, external tools, and sequential computation. The corresponding unit of evaluation must therefore expand from individual model performance to the reliability of the integrated system, a distinction that becomes central in the sections that follow. This evolution from task-specific prediction towards increasingly integrated AI capabilities is summarized in Figure 1.

The conceptual trajectory of healthcare AI from task-specific predictive models to representation learning and foundation models, followed by the emergence of multimodal and generative capabilities and their integration into agentic systems. The progression represents an expansion of model scope and system-level capability rather than the replacement of earlier paradigms. The clinical and translational characteristics of these AI paradigms are summarized in Table 1.

## 3. Predictive AI in Diagnosis: From Performance to Clinical Utility

Predictive AI is one of the most established uses of artificial intelligence in medicine. It has been applied to disease risk estimation, prognosis, future clinical events, and treatment response, often using complex and heterogeneous clinical data. Its advantage lies in the ability to capture patterns that may be difficult to represent with conventional clinical scores. However, greater model complexity does not necessarily lead to better clinical performance. Machine-learning models have not consistently outperformed well-specified statistical approaches, particularly in studies with limited sample sizes, inadequate validation, or high risk of bias [6]. The key question is therefore no longer whether a model can predict accurately but whether its predictions are reliable across settings, well calibrated, clinically actionable, and capable of improving care.

### 3.1. Risk Prediction and Stratification

Risk prediction is a natural application of clinical AI because many medical decisions depend on estimating the likelihood of future events. Machine-learning models have been used to predict deterioration, complications, recurrence, hospitalisation, mortality, and other clinically important outcomes using electronic health records, laboratory data, imaging-derived features, physiological signals, and longitudinal information [29,30,31,32,33].

These models can capture nonlinear relationships and interactions across large numbers of predictors, which may be difficult to represent in conventional risk scores based on a limited set of predefined variables. However, greater complexity does not necessarily provide greater predictive value. Comparative studies have shown that the apparent advantage of machine learning over logistic regression often decreases when methodological quality, sample size, and risk of bias are taken into account [6]. The relevant comparison is therefore not between newer and older algorithms but between competing approaches evaluated under similar conditions.

High discrimination alone is insufficient for clinical use. A model may rank patients accurately while still producing poorly calibrated risk estimates or failing to identify clinically useful decision thresholds. A predictive model becomes clinically useful when its estimated probabilities are adequately calibrated to observed outcomes and its outputs meaningfully inform actionable clinical decisions. This requirement is particularly important when model outputs influence monitoring intensity, escalation of care, or allocation of limited healthcare resources.

### 3.2. Diagnostic Prediction: Disease Detection and Differential Diagnosis

Diagnostic AI has provided some of the clearest demonstrations of high-dimensional pattern recognition in medicine. Meta-analytic evidence shows that deep-learning systems can achieve high diagnostic performance across several image-intensive specialties, including radiology, ophthalmology, pathology, and endoscopy [7,34,35,36]. These findings established technical feasibility but also exposed an important limitation of the early literature: performance was frequently estimated in retrospective, curated datasets that differed substantially from the populations in which the systems would ultimately be used [7].

The distinction between benchmark accuracy and clinical diagnostic performance is consequential. Disease prevalence, case spectrum, acquisition protocols, reference standards, and referral pathways can all alter model behaviour. Comparisons between an algorithm and clinicians on a static test set therefore provide limited evidence about how the same system will perform when confronted with consecutive patients, uncertain presentations, workflow constraints, and changing data distributions.

Diagnostic studies should specify the target condition and relevant alternatives, care setting, intended population, index test, reference standard, and the model’s role as a replacement, triage, add-on, or second-reader test. A screening system may prioritize sensitivity and manageable referral burden, whereas a confirmatory aid may require high specificity and calibrated probabilities. A differential-diagnosis system must rank clinically plausible alternatives, identify discriminating evidence, and signal when available data are insufficient. False-negative and false-positive consequences should therefore be evaluated at clinically selected thresholds rather than inferred from AUROC alone [7,8,9,10].

External and prospective studies provide a more stringent test. Clinical validation of diabetic retinopathy screening in African populations demonstrated the importance of evaluating algorithms beyond the populations in which they were initially developed [37]. Similar principles have been examined in externally validated systems for pulmonary nodule malignancy [38], pancreatic cancer [39], coronary calcium assessment [40], and AI-assisted pathology [41,42]. Collectively, these studies mark a shift from asking whether an algorithm can recognise disease towards asking whether diagnostic performance survives changes in population and clinical setting.

The relevant endpoint is therefore no longer a marginal improvement in AUROC or sensitivity alone. For mature diagnostic AI, the evidentiary standard should increasingly include independent validation, representative case spectra, clinically relevant thresholds, and evaluation of how algorithmic information modifies human decisions.

For practical implementation, each output must be linked to a prespecified diagnostic action, such as repeat testing, specialist referral, biopsy, treatment initiation, or safe discharge, and to a failure or escalation pathway. Prospective diagnostic studies should recruit consecutive or otherwise representative patients, use blinded and clinically credible reference standards, retain indeterminate and missing cases in the analysis, and report diagnostic yield, time to diagnosis, additional testing, net benefit, downstream harms, and subgroup performance. This test-to-action chain distinguishes diagnostic validity from diagnostic utility.

### 3.3. Prognostic Stratification After Diagnosis and Treatment-Response Prediction

Prognostic AI addresses a different clinical question: not whether disease is present, but what is likely to happen after diagnosis. Predictive models have been developed to estimate survival, relapse, disease progression, complications, and functional recovery across a wide range of clinical conditions [30,31,32,33]. Oncology has been a particularly active area for AI-based prognostic modelling because of the availability of large, longitudinal, and increasingly multimodal clinical datasets. Prognosis refers to the expected course or probability of future clinical outcomes after a defined index point, such as diagnosis or treatment initiation. In clinical studies, the clinical outcome after a clinical decision may change with time, follow-up information may be incomplete and study endpoints may be defined differently by studies and institutions. Therefore, a good predictor of a negative clinical outcome does not necessarily provide insight into the reasons for the prediction or the potential effects of any clinical intervention to change the predicted outcome. A predictor of treatment response must distinguish between predicting poor outcome for a given patient and predicting that a given patient is more likely to benefit from one treatment vs. another. In other words, association of a clinical parameter with an outcome does not necessarily provide evidence for its therapeutic use.

While AI-based predictive models can be of great value to precision medicine by enabling decisions in the context of large amounts of data, their inclusion in treatment decisions requires more than good prognostic performance of a model.

### 3.4. External Validation and Generalisability

External validation is a critical step between model development and clinical use. Internal validation can estimate performance within the development setting, but it cannot show whether a model will perform similarly in different hospitals, populations, devices, or periods of clinical practice. External validation addresses this question by testing the transportability of model performance beyond the data on which it was developed [8].

This matters because clinical environments are rarely identical. Disease prevalence and referral patterns vary across regions, patient populations differ in baseline risk, and changes in clinical practice can alter treatment and coding. Differences in scanners, laboratory assays, and electronic health-record systems may further shift the data presented to the model.

Performance deterioration under these conditions is not an exceptional failure of AI but an expected consequence of dataset shift.

Prospective and multicentre evaluations provide increasingly important evidence of this transition. Studies have evaluated AI systems across independent populations for diabetic retinopathy [37], pulmonary nodules [38], left-ventricular systolic dysfunction [43], pancreatic cancer [39], coronary calcium scoring [40], developmental screening [44], diabetic-retinopathy programmes involving tens of thousands of patients [45], acute kidney injury [46], and breast-cancer prognostication [47]. Their collective importance lies less in the individual clinical domains than in demonstrating that model performance must be re-established when the data-generating environment changes.

Generalisability also cannot be judged by discrimination alone. AUROC describes ranking ability but does not establish whether a predicted probability corresponds to the observed event rate. Calibration is therefore essential whenever predictions support threshold-based decisions. Evaluation should consequently extend beyond accuracy, sensitivity, specificity, and AUROC to include calibration and, where appropriate, decision-analytic measures that quantify whether predictions improve decisions at clinically relevant thresholds [9].

External validation should thus be interpreted as a minimum requirement for translation rather than evidence of clinical effectiveness. A transportable model may still fail to improve care.

### 3.5. From Prediction to Diagnostic Utility and Patient Benefit

The most consequential gap in predictive AI lies between knowing that a model predicts accurately and knowing that using the model benefits patients. These are distinct evidentiary questions.

An externally validated model can remain clinically ineffective if its predictions arrive too late, duplicate information already available to clinicians, generate excessive alerts, encourage inappropriate intervention, or cannot be incorporated into existing workflows. Conversely, a model with only modest improvements in discrimination may be valuable if it reliably identifies a decision threshold at which management changes and patient benefit outweighs harm. The clinical evaluation is not a single validation step but rather a step in a chain of steps that need to be taken to determine the potential of AI in clinical settings (see Table 2). Technical evaluation establishes whether the model can reliably capture the intended signal, whereas external validation assesses whether performance is transportable to independent populations and settings. Workflow evaluation examines interactions among the AI system, clinicians, patients, and existing clinical processes, including potential safety and human-factor risks [10]. Clinical-impact studies subsequently determine whether AI-assisted care improves clinically meaningful processes or patient outcomes, ideally through comparative or randomized evaluations where appropriate [21,22]. This final transition remains comparatively underdeveloped. Prospective evaluations such as large-scale diabetic-retinopathy screening demonstrate that AI systems can be assessed under operational conditions [45], while deployment studies in pathology have begun to examine AI within clinician-facing diagnostic workflows [41,42]. Randomised evidence provides an even stronger test. In the HYPE trial, a machine-learning-derived early-warning system for intraoperative hypotension was evaluated against standard care, illustrating the methodological shift from assessing predictive accuracy to assessing the consequences of model-guided intervention [20].

The distinction is central to the interpretation of progress in predictive AI. A higher AUROC is not synonymous with better medicine. Clinical maturity requires evidence that predictions remain reliable outside the development dataset, provide information that changes an appropriate decision, and produce benefits that exceed the harms and costs introduced by the system. Evaluation must also continue after deployment because changes in populations, clinical practice, and data-generating systems can alter performance over time; contemporary consensus guidance therefore treats monitoring as part of the AI lifecycle rather than an endpoint after implementation [23].

Predictive AI has the most established translational evidence base, extending from retrospective and external validation to prospective and clinical-impact evaluation in selected applications. However, the level of evidence remains heterogeneous across clinical domains.

## 4. Generative AI in Diagnosis: From Language Models to Clinical Copilots

Generative artificial intelligence has altered the functional scope of clinical AI by moving beyond predefined outputs towards the synthesis, transformation, and contextualisation of medical information. Unlike conventional clinical prediction models designed to estimate a predefined class, probability, or risk endpoint, LLMs generate context-dependent sequences and can support a broader range of information-intensive clinical tasks. In clinical settings, these capabilities can support multistep tasks such as extracting relevant information, generating differential diagnoses, summarizing patient records, and presenting medical information in a form that is easier for patients to understand. They can be used to create clinical documents and respond to subsequent questions as more information becomes available. Early demonstrations established substantial medical knowledge within large pretrained models [48], but subsequent clinical studies have made clear that knowledge retrieval and clinically reliable reasoning are not equivalent. The relevant translational question is therefore no longer whether LLMs can generate plausible medical text but whether their outputs remain correct, context-sensitive, and useful when incorporated into human clinical reasoning and care workflows.

### 4.1. Clinical Reasoning Beyond Benchmark Performance

Medical examinations and vignette-based benchmarks initially provided convenient measures of LLM capability, but they test only a restricted subset of clinical reasoning. Real clinical decisions require sequential information gathering, interpretation of incomplete or conflicting evidence, adherence to guidelines, adjustment to changing context, and recognition of uncertainty. When these demands are incorporated into evaluation, performance becomes substantially less uniform.

Hager and colleagues evaluated state-of-the-art LLMs across 2400 real patient cases in a simulated clinical decision environment and found important deficiencies in diagnosis, guideline adherence, interpretation of laboratory information, and sensitivity to the amount and ordering of clinical information [49]. This distinction is important because fluent explanations can create an impression of reasoning even when the underlying inference is unstable.

At the same time, more recent systems demonstrate that generative models can contribute meaningful diagnostic information under appropriately constrained conditions. Prompt structures that require explicit diagnostic reasoning can expose intermediate clinical considerations [50], while newer models have shown improved performance in differential-diagnosis generation [51]. The resulting picture is therefore neither one of clinical equivalence nor of simple failure. LLM capability is highly task-dependent, and performance measured in isolated question-answering tasks cannot be assumed to generalise to longitudinal or high-consequence decisions.

### 4.2. Human-LLM Collaboration Rather than Autonomous Diagnosis

The clinically relevant unit of evaluation is increasingly the human–AI team rather than the model in isolation. This change is supported by emerging comparative and randomised evidence.

In a randomised clinical trial involving 50 physicians, access to an LLM did not significantly improve diagnostic-reasoning scores compared with conventional resources, despite the LLM performing strongly when evaluated alone [52]. This apparently paradoxical result is instructive. A capable model does not automatically produce a more capable clinical team. Effective augmentation depends on how recommendations are presented, when the system is queried, whether clinicians recognise incorrect suggestions, and how much weight is assigned to model outputs.

Generative AI should be evaluated not only by whether it produces the correct answer, but also by how it influences clinical reasoning. Important effects on information search, on framing the diagnostic problem, on dealing with uncertainty, on time to decide, on cognitive load, and on automation bias need to be considered. The clinically relevant question is therefore not whether an AI model can independently reach the correct diagnosis, but whether its use improves clinicians’ diagnostic reasoning and accuracy while reducing error. Direct model-versus-clinician comparisons alone provide limited insight into the clinical value of human–AI collaboration. Evaluation should instead determine which components of clinical reasoning can be effectively augmented by AI, which should remain under clinician control, and where independent verification is required.

For diagnostic use, LLMs should not be evaluated only on the correctness of a final answer. They should be tested on problem representation, breadth and prioritization of the differential diagnosis, identification of discriminating findings, selection of the next diagnostic tests, and calibration of uncertainty. A useful copilot should surface dangerous alternatives, ask for missing information, resist false premises, and abstain or escalate when evidence is insufficient. Studies should separately score omission of critical diagnoses, unsupported additions, harmful test recommendations, and clinician overreliance [49,50,51,52,53].

### 4.3. Generation as a Clinical İnformation İnterface

The near-term value of generative AI may be greatest in tasks centred on processing and communicating clinical information rather than making diagnoses. LLMs can summarise longitudinal records, draft clinical notes, translate technical language, retrieve information from institutional guidance, and adapt information for different users. These tasks make practical use of generative capabilities while preserving a clear role for human review.

Patient communication illustrates both the potential and the limitations of this approach. An evaluation of AI-assisted simplification of hospital discharge documentation found that generated summaries improved readability for patients; however, clinician review identified factual inaccuracies, omissions, and potentially safety-relevant errors [54]. Improved comprehensibility should therefore not be interpreted as equivalent to improved factual reliability.

Performance can also vary substantially across clinical tasks. In an evaluation using clinical vignettes, LLM performance was better for final diagnosis than for initial differential diagnosis or management [53]. Aggregate accuracy may therefore obscure weaknesses that become important when a model is used at different stages of clinical care.

More recent work has begun to evaluate generative AI in bounded clinical settings. PEACH, a perioperative chatbot grounded in 35 institutional protocols, was tested through silent deployment using real-world clinical interactions and achieved high guideline-concordant accuracy with few hallucinations, although its effect on patient outcomes remains unknown [55]. Such systems may offer a more realistic near-term model for clinical generative AI: constrained copilots operating within defined knowledge and workflow boundaries, with clinicians retaining oversight.

### 4.4. Hallucination, Factuality, and Context-Dependent Failure

Generative AI introduces a different type of failure from conventional predictive models. A predictive model may assign the wrong probability or class, whereas an LLM can produce incorrect information within a fluent and convincing explanation. This makes factual errors harder to recognize and potentially more consequential in clinical settings.

Hallucinations can result from missing knowledge, ambiguous prompts, unsupported inference, retrieval errors, conflicting context, or manipulated inputs. Standard benchmarks may not capture these vulnerabilities. Alber and colleagues showed that introducing a small amount of medical misinformation into training data increased harmful outputs without substantially affecting conventional benchmark performance [56]. Similarly, in physician-validated simulated cases containing false information, LLMs often elaborated on fabricated clinical details; mitigation prompting reduced but did not eliminate this behaviour [57].

In clinical evaluation of factual reliability of AI systems, it is important to test their ability to reject incorrect premises, to distinguish between evidence and assertion, and to express uncertainty. While retrieval augmentation, domain-specific grounding, constrained output spaces, and verification can all reduce errors, none of them on their own is sufficient. Factual reliability is a property of the overall information pathway consisting of the model, the retrieved evidence, the prompting, the user interaction with the system and the surrounding clinical system.

### 4.5. From General-Purpose Generation to Bounded Clinical Copilots

The evidence increasingly supports a distinction between general capability and clinical readiness. LLMs can demonstrate sophisticated medical knowledge and, in selected tasks, high diagnostic performance; however, the same models can fail under realistic information sequences, propagate false premises, or provide outputs that clinicians do not use effectively [49,52,56,57]. Clinical deployment therefore requires narrowing rather than simply expanding the model’s operating space.

A clinical copilot is defensible only when it is bounded to a clearly specified task, relies on explicitly defined information sources, interacts transparently with the EHR or institutional knowledge base, appropriately represents uncertainty, maintains an auditable record of its outputs and supporting evidence, and incorporates predefined points of clinician oversight and control. Recent studies have begun to evaluate bounded clinical LLMs under real-world or workflow-specific conditions, including perioperative care. For example, protocolized deployment of a simple, task-focused LLM in the form of a decision support tool yielded accurate and useful results [55]. In emergency care, language-model systems have also been developed around clinical records and workflow-specific tasks rather than unrestricted conversational use [58].

This shift has an important implication for the trajectory of generative AI in medicine. Progress should not be judged primarily by increases in parameter count, examination scores, or the apparent sophistication of generated responses. More meaningful advances will come from systems that reliably retrieve the right evidence, preserve clinically relevant context, expose uncertainty, support rather than distort human reasoning, and operate safely within clearly specified workflows. Clinical-system evaluation should therefore extend beyond model-level accuracy to include error propagation, abstention and escalation, clinician override, and the downstream consequences of incorrect outputs.

Generative models are also becoming increasingly capable of jointly processing text and other biomedical data. Early multimodal systems have shown the feasibility of combining clinical language with images for tasks such as dermatological assessment [59] and radiology-report generation [60]. SkinGPT-4, for example, was evaluated on real clinical dermatology cases with specialist input, whereas clinician-vision–language collaboration in radiology demonstrated both potential utility and clinically significant errors in generated reports. Because that transition changes the nature of both the available information and the possible failure modes, multimodal AI is considered separately in the next section rather than treated as an incremental extension of LLMs here.

Evidence for generative AI remains predominantly benchmark-based or retrospective, with comparatively limited prospective, workflow, and clinical-impact evaluation. Its clinical maturity therefore remains below that of established predictive applications.

## 5. Multimodal AI for Diagnostic Evidence Integration

Multimodal artificial intelligence extends clinical AI beyond the analysis of individual data types by integrating complementary information within a unified computational framework. This approach is particularly relevant to medicine, where diagnosis, prognosis, and treatment decisions routinely depend on the joint interpretation of heterogeneous evidence rather than any single measurement. However, multimodality should not be regarded as an objective in itself. Its clinical value depends on whether integration captures complementary information, remains robust when data are incomplete, and provides measurable benefit over well-performing unimodal approaches. This section examines these principles through multimodal patient representations, vision–language models, incremental clinical value, and the challenges associated with data fusion and missing modalities.

### 5.1. From İsolated Signals to İntegrated Patient Representations

In many clinical situations, decisions are not made based on the analysis of single data sources, such as imaging, lab results, EHRs, physiological signals, histology or molecular data from tumors. Instead, all of these sources contain partly redundant and partly complementary information on the patient. Instead of analyzing each of these sources for prediction in isolation, they can be modeled jointly by multimodal AI approaches. The value of a particular modality does not necessarily increase with the number of input modalities. Rather, its value is determined by whether it provides additional information that changes discrimination, calibration, or even clinical decisions made by the best unimodal model.

Primary studies from a number of diseases are illustrated in this section, including joint histology–genomic modelling across a number of cancer types to identify prognostic information [61], while integration of radiology, pathology, genomics, and clinical variables improved risk stratification in high-grade serous ovarian cancer [62]. Flexible multimodal frameworks have also combined routinely collected clinical information with neuroimaging for dementia assessment [63] and radiographs with clinical variables for osteoarthritis progression [64]. These studies move the field closer to the way clinicians actually reason, but they also expose a central methodological requirement: multimodal models should be judged against strong unimodal comparators, not against weak or historically convenient baselines.

### 5.2. Vision–Language Models as a Clinically İmportant Multimodal İnterface

Vision–language models (VLMs) extend multimodal learning by aligning images with natural language, enabling a model to interpret visual findings in clinical context and, in some settings, generate explanatory text. Their appeal is strongest in specialties in which images and narrative interpretation are inseparable. Transformer-based integration of chest radiographs and clinical parameters has shown that contextual non-imaging information can improve diagnostic performance over single-modality approaches [65]. In dermatology, SkinGPT-4 combined images with clinical concepts and physician notes and was evaluated on real clinical cases [59]. In pathology, in-context learning with a multimodal model has shown that image classification can be adapted to new cancer tasks with few examples and without conventional task-specific retraining [66].

However, multimodal fluency should not be equated with clinical reliability. In radiology report generation, clinician evaluation of Flamingo-CXR showed substantial potential for image-text assistance but also clinically significant errors in both AI-generated and human reports [60]. More recent benchmarking in emergency and critical care similarly indicates that VLM performance remains sensitive to task and model choice [67]. The most defensible near-term role is therefore not autonomous image interpretation, but context-aware assistance in which generated conclusions remain auditable against source images and clinical data.

### 5.3. Does Multimodality Provide İncremental Clinical Value?

The decisive question is whether multimodal integration adds clinically meaningful information. Several well-designed primary studies support this premise, but the magnitude of benefit is heterogeneous. In pulmonary embolism detection, fusion of CT pulmonary angiography with EHR data outperformed imaging-only and EHR-only models [68]. Multimodal chest-radiograph models similarly benefited from combining imaging with clinical parameters across diagnostic tasks [65]. In oncology, complementary information from imaging, pathology, and molecular data has improved prognostic modelling and risk stratification [61,62]. MultiSurv further demonstrated the feasibility of integrating clinical, imaging, and multiple omics modalities for pan-cancer survival prediction [69].

Yet a statistically superior multimodal model is not automatically a clinically superior model. Small absolute gains may not justify additional tests, data acquisition, computational complexity, or delayed decision-making. Apparent benefit can also reflect information leakage, unequal preprocessing, or a weak unimodal comparator. Incremental value should therefore be demonstrated using identical cohorts and endpoints, strong modality-specific baselines, uncertainty estimates, calibration, and where possible external or prospective validation. The relevant endpoint is not multimodal superiority per se, but whether the added information is sufficiently large, reproducible, and actionable to alter care.

For diagnostic use, incremental value should be expressed in terms that reflect the care pathway: change in sensitivity or specificity at the selected threshold, reclassification, diagnostic yield, avoided or added tests, time to diagnosis, and net benefit. The reference standard should be independent of the modalities supplied to the model whenever possible to reduce incorporation bias, and temporal alignment should prevent future information from leaking into the diagnostic prediction. If a required modality is missing or degraded, the system should provide a validated fallback, an uncertainty warning, or abstain rather than issue an unqualified conclusion [61,62,63,64,65,66,67,68,69,70].

### 5.4. Fusion and the Missing-Modality Problem

How modalities are combined matters because clinical data differ in structure, timing, reliability, and availability. Early fusion may obscure modality-specific information, whereas late fusion can miss interactions between data sources. More flexible approaches can address some of these limitations, but no fusion strategy eliminates a common problem in clinical practice: multimodal data are often incomplete.

Models trained only on complete cases may perform poorly when one or more modalities are unavailable at deployment. MultiSurv was designed to handle missing values and missing modalities [69] while more recent attention-based approaches use modality-specific representations and contrastive learning to preserve performance when inputs are incomplete [70]. Missing data should therefore be treated as part of the clinical setting rather than simply as a preprocessing problem. Evaluation should reflect realistic patterns of modality availability and consider that missingness itself may carry clinically relevant information. Evaluation should further determine whether integration failures or discordant inputs are detected and contained rather than propagated into downstream clinical decisions.

### 5.5. Clinical İmplications

Multimodal AI is most useful when it combines genuinely complementary information and remains reliable when some data are missing. The goal should not be to add as many modalities as possible, but to include those that provide clear incremental value. Each added data source should improve clinically relevant performance and the model should remain robust when that source is unavailable or degraded. This principle is particularly relevant for precision oncology, neurological disease, acute imaging, and longitudinal risk prediction, where clinically meaningful information is distributed across heterogeneous sources [61,62,63,65,68,69,70,71].

The evidence to date supports multimodal AI as an important direction for clinical decision support, but not as a universal replacement for unimodal systems. A well-calibrated single-modality model may remain preferable when additional data are costly, inconsistently available, or only marginally informative. Clinical maturity will therefore depend less on demonstrating that modalities can be fused than on showing that integration provides reproducible incremental value under the incomplete, heterogeneous, and shifting conditions of routine care. Representative primary studies illustrating the clinical value and remaining limitations of multimodal AI are summarized in Table 3.

Multimodal AI is supported mainly by retrospective studies, with external validation emerging in selected applications. Evidence of prospective performance and incremental clinical benefit over strong unimodal approaches remains limited.

## 6. Agentic AI in Diagnostic Workflows: From Assistance to Goal-Directed Clinical Action

In this Review, a system is considered agentic when it pursues an explicit goal through stateful and iterative interaction with its environment, supported by the following operational capabilities: goal-directed planning, maintenance of context or memory, selection of tools or actions, evaluation of intermediate observations, and adaptation of subsequent actions. The degree of autonomy is considered separately according to the extent of human control and oversight. Accordingly, the use of multiple agents, external tools, or a multi-step LLM pipeline alone is not sufficient for a system to be classified as agentic.

Agentic AI extends the trajectory of clinical artificial intelligence from information generation toward systems capable of coordinating multi-step tasks within defined goals and constraints. The relevance of this development to healthcare is not merely that it enables more autonomous behavior of computer programs. Of much greater relevance is the capability of such programs to link their reasoning to external knowledge, to application-specific tools and to the step-wise execution of workflows. Clinical evaluation of such systems therefore needs to be expanded from being centered on a single output to covering the entire decision–action path that such systems are able to produce. This section examines the progression from bounded clinical agents to multi-agent architectures and human–AI collaboration, together with the associated governance requirements.

### 6.1. From Generative Assistance to Agency

Agentic AI extends generative systems from producing individual responses to pursuing goals through planning, tool use, and sequential action. In healthcare, an agent may retrieve additional evidence, use specialised models or clinical calculators, revise its approach, and coordinate multiple steps within a workflow. Evaluation must therefore consider not only the quality of a single response, but also the sequence of decisions and actions taken to reach an outcome. This creates new opportunities for clinical workflow support, while also increasing the ways in which errors can arise and propagate.

Early primary studies suggest that bounded agents can perform clinically meaningful tasks when their objectives and tools are tightly specified. Autonomous oncology decision support has been developed and validated around structured clinical reasoning [72], while LLM-based agents have been tested for evidence-based medicine [73], radiotherapy planning [74], order-set optimisation [75], and disease-specific diagnostic or treatment planning [76]. These applications are more informative than generic conversational benchmarks because they require the system to operate within a defined clinical process rather than merely produce plausible text.

### 6.2. Planning, Tool Use, and Multi-Agent Reasoning

The principal advantage of an agentic architecture is decomposition. Complex clinical tasks can be divided into information extraction, evidence retrieval, differential generation, verification, and recommendation, with each step assigned to a specialised tool or agent. Multi-agent designs extend this principle by allowing several role-specific components to critique or refine one another. CARE-AD, for example, used a multi-agent LLM framework to analyse longitudinal clinical notes for Alzheimer’s disease prediction [77]. Similar architectures have been reported for neuro-ophthalmic diagnosis and personalised treatment planning [76] and for optimisation of clinical order sets [75].

However, additional agents do not inherently produce additional clinical value. Decomposition can improve traceability and error checking, but it can also introduce correlated reasoning, duplicated errors, communication failures, and greater computational cost. Comparisons of rule-based, single-agent, and multi-agent systems are therefore particularly important because they test whether orchestration itself contributes value rather than simply increasing system complexity [78]. The appropriate comparator for an agentic system is not an unassisted language model alone, but the strongest simpler workflow capable of performing the same task.

In diagnostic workflows, an agent may assemble longitudinal evidence, generate and reprioritize differential diagnoses, select or call diagnostic tools, and route referrals. Because an early patient-matching, retrieval, or anchoring error can propagate through every subsequent step, validation should evaluate each transition: data retrieval, patient identification, problem representation, differential generation, test selection, interpretation, and escalation. Explicit stop rules are required before invasive, costly, or otherwise high-consequence actions [72,73,74,75,76,77,78,79,80].

### 6.3. From Decision Support to Workflow Execution

Agentic systems become clinically distinctive when they move beyond recommendation towards workflow execution. This includes selecting and calling external tools, retrieving patient-specific information, generating intermediate artefacts, and determining the next action from previous results. A feasibility study of LLM agents for radiotherapy planning illustrates this transition: the model was embedded within a sequence of planning operations rather than evaluated only on textual knowledge [74]. Likewise, autonomous analysis of curated oncology data and agent-based clinical decision systems indicate a movement towards systems that coordinate multiple analytic steps before producing an output [72,79].

This transition should nevertheless remain bounded by the reversibility and consequence of the action. Drafting an order set for clinician approval and autonomously placing an order are not equivalent levels of agency. Clinical deployment therefore requires explicit action permissions, tool whitelisting, audit logs, provenance of retrieved evidence, and predefined escalation points. Human oversight is most meaningful when it is positioned before high-consequence or irreversible actions rather than appended as a nominal final review.

### 6.4. Safety and Evaluation of Agentic Systems

Agentic AI changes the object of safety evaluation. Simply assessing accuracy at the final output can be insufficient, since a seemingly correct output can hide an unsafe sequence of actions, and an early error in patient identity or retrieval can propagate to affect all subsequent actions. Instead, evaluation should assess the full completion of a task, the intermediate reasoning and all tool calls made during task completion, error recovery, adherence to specified constraints, patient identity, robustness to incorrect information, and the system’s ability to stop and/or escalate to a human as needed. Note that recent work has demonstrated silent failure of clinical natural language processing agents on patient identity, highlighting the insufficiency of answer-level evaluation for systems that have access to patients’ longitudinal records and/or to execute specific tools to support clinical tasks [80].

Safety must also be assessed at the system level. Multi-agent architectures introduce internal communication channels and new privacy and security boundaries; autonomous agents can amplify small upstream errors through repeated actions. For clinical use, the central question is therefore not whether an agent can complete a workflow under ideal conditions, but whether it fails detectably and recoverably when information is missing, contradictory, or incorrect. Prospective evaluation should report not only clinical performance but intervention frequency, override behaviour, failure severity, and the consequences of erroneous tool use.

### 6.5. Clinical Readiness

Agentic AI should be viewed as a relatively new and emerging layer of clinical automation rather than a fully developed autonomous clinician. The strongest near-term use cases for Agentic AI are bounded, auditable workflows—such as goal, tool, data source, action sets defined by humans—in which complex information processing is simplified and otherwise discrete AI applications are integrated by the agent. There is much less evidence to date to support the autonomous management of clinical objectives and their implementation by the system alone.

Clinical readiness therefore depends on controllability. Actions should be clearly bounded, the evidence supporting each action should be traceable, tool failures should be detectable, and predefined pathways for escalation to a human clinician should be available. As AI systems acquire greater capacity to perform actions within clinical workflows, their evaluation must extend beyond response quality to include the safety and reliability of the end-to-end clinical process. As the copilots of today’s AI systems transition to full agents that take actions, as opposed to simply generating information, validation of such systems will transition from assessing the quality of their responses to assessment of the safety of the end-to-end clinical workflow. These are issues of governance that go beyond assessing the performance of individual models, and are therefore discussed in the following section. Representative clinical studies evaluating different forms and applications of agentic AI are summarized in Table 4.

Agentic AI remains at an early translational stage, with evidence largely derived from proof-of-concept and task-level evaluations. Independent validation, prospective workflow studies, and evidence of clinical impact are currently limited.

## 7. Diagnostic and Clinical Applications Across Healthcare

Artificial intelligence is being introduced into the clinical setting in different ways depending on the medical discipline. For image-based disciplines such as dermatology, the translation of computer vision into clinical applications for diagnostic assistance is slowly being developed and evaluated. In contrast, data-rich disciplines are increasingly integrating heterogeneous sources, including patient records, physiological signals, laboratory findings, molecular profiles, and patient-generated health data, and are being used for screening, diagnostic, prognostic, therapeutic, procedural, alerting and management purposes of chronic diseases over long periods of time.

Measures of model performance alone do not establish the clinical importance of AI applications. Even highly accurate models may provide limited clinical benefit if they reproduce information already recognized by clinicians, deliver outputs too late to influence decision-making, or generate additional workload that outweighs their potential utility. Table S1 represents the various stages and forms of clinical translation that have been achieved in various clinical areas rather than representing the highest performing models for each clinical area.

Across specialties, AI occupies four diagnostically distinct roles: screening or triage, lesion or disease detection, differential diagnosis or etiologic classification, and prognostic or treatment-response stratification. These roles have different prevalence, reference-standard, operating-threshold, and downstream-action requirements and should not be compared using a single accuracy metric. Table S1 therefore maps each study to its AI task, clinical application, and stage of evidence rather than ranking algorithms by performance.

The clearest diagnostic translation pathways are seen when the AI output is connected to a defined next action: recall or referral in mammography and retinal screening [81,82,83], real-time lesion detection in colonoscopy [84,85], pathologic or intraoperative classification [41,86], etiologic dementia differentiation [87], and work-up of prostate cancer, aneurysm, fracture, melanoma, or pediatric respiratory disease [88,89,90,91,92,93]. These examples show why evaluation should progress from accuracy to prospective diagnostic yield, additional testing, time to diagnosis, decision change, and downstream outcomes.

Studies on clinical AI applications that are close to clinical practice show that clinical AI is not following a uniform translational trajectory. In some applications, the distance between algorithmic output and an actionable clinical decision is relatively short.

However, the vast majority of clinical AI applications require a substantially longer translational pathway to deliver clinical utility. For example, many prognostic models developed for use in oncology, nephrology, psychiatric and neurological care of chronic conditions may perform well at risk stratification but lack evidence of how the AI generated information results in changes to treatment or patient outcomes. Clinically mature AI applications are those that deliver information that is incremental, actionable, on time and most importantly of benefit within the specific clinical care pathway. The major data modalities underpinning AI applications across clinical specialties are summarized in Table 5. The table provides a conceptual mapping of commonly used data sources rather than a quantitative ranking of modality prevalence. Modalities increasingly coexist within multimodal systems, and their clinical relevance depends on the task, care setting, and availability of complementary information.

## 8. Trustworthy Clinical AI: Safety, Equity, Transparency, and Governance

The clinical value of AI-based decision support extends beyond predictive accuracy. Such a system must also support the clinician in decision making about diagnosis and/or treatment, in setting priorities and in monitoring patients. Safe implementation requires a sociotechnical system that remains reliable across relevant patient populations and clinical settings, provides appropriate transparency and traceability, and supports clear accountability.

For diagnostic AI, trustworthiness requires explicit management of false-negative and false-positive harms, indeterminate outputs, subgroup-specific performance, abstention and escalation criteria, and responsibility for the final interpretation. Prevalence drift requires particular attention because it changes positive and negative predictive values even when sensitivity and specificity appear stable [23,94,95,96,97,98,99,100,101].

### 8.1. Trustworthiness İs a System Property

Traditionally, AI has been developed in a retrospective fashion and then applied to clinical settings. As the technology begins to be used in more clinical scenarios, however, performance is not the only consideration for defining quality. A clinically trustworthy system must remain safe and useful for different populations, in different settings, over time. Importantly, all outputs must be traceable and all individuals involved in decision-making for critical clinical decision points must be clearly identified. The FUTURE-AI consensus framework outlines a set of characteristics that define trustworthy healthcare AI (fairness, universality, traceability, usability, robustness, and explainability) that must be embedded across the system lifecycle [23]. Importantly, trustworthiness is not an after-thought additional metric; it is a property of the data, model, interface, workflow, institution, and monitoring system.

While aggregate accuracy may hide failures in individual cases, a number of other types of failures are possible, such as systematic failure in certain subgroups of patients, using inappropriate stand-ins for important clinical variables, encouraging over-reliance, and failing after deployment. Governance must consequently focus on how performance is distributed and maintained, not simply on whether a model crossed a prespecified benchmark before release.

### 8.2. Bias, Fairness, and Health Equity

Clinical AI can reproduce inequities already embedded in healthcare data and can create new disparities through differential model performance. A widely used population-health algorithm was shown to underestimate the health needs of Black patients because healthcare cost was used as a proxy for illness; correcting the proxy substantially increased the proportion of Black patients identified for additional care [102]. In medical imaging, chest-radiograph classifiers have demonstrated systematically higher underdiagnosis rates in underserved groups, including Black and Hispanic patients and patients with lower socioeconomic status [95].

Fairness cannot therefore be reduced to demographic balance in the development dataset. Models can encode sensitive attributes even when those variables are not explicitly supplied. Deep learning systems can infer self-reported race from multiple medical imaging modalities with high accuracy, including under transformations that obscure obvious anatomical or acquisition-related explanations [96]. Removing protected variables is consequently not a sufficient mitigation strategy. Evaluation should examine clinically relevant error rates, calibration, and downstream consequences across prespecified and intersectional subgroups, while recognising that statistical fairness criteria may conflict and must be selected according to the clinical use case.

### 8.3. Explainability, Transparency, and Appropriate Reliance

Explainability is often seen as the key to winning users’ trust for AI systems. However, explaining a model’s behavior is not enough; the explanation must also be accurate. Most methods for explainable AI, including saliency maps, feature-attribution methods, and natural-language rationales, aim to make a system more interpretable than it actually is. The clinical objective of calibrated reliance therefore is to support users by describing the system’s intended use, limitations, degree of uncertainty, and evidence in order to enable them to decide whether to trust the system, to verify it, or even to reject it.

Human–AI interaction studies reveal the complexities of how human clinicians might be influenced by AI in clinical decision-making environments. While clinicians benefit from assistance provided by AI systems, incorrect information can mislead them. Moreover, clinicians can modify their clinical decisions in negative ways when they perceive an AI system to be authoritative [97]. Appropriate reliance is bidirectional. Clinical performance may be compromised not only by over-reliance on incorrect AI recommendations but also by algorithm aversion or under-reliance, particularly when clinicians perceive algorithmic advice as less trustworthy or as constraining professional judgment. Effective human–AI collaboration therefore requires calibrated reliance rather than either uncritical acceptance or systematic rejection of AI-supported recommendations [97,103]. Conversely, collaborative evaluation in dermatology has shown that human and AI performance depends on the interaction design and the clinician’s ability to integrate algorithmic information [98]. Transparency should therefore include provenance, versioning, data lineage, intended population, operating thresholds, and known failure modes rather than relying on post hoc visual explanations alone.

### 8.4. Privacy, Security, and Robustness

Clinical AI creates security risks at both the data and model levels. Training data may contain identifiable or inferable patient information, while deployed models can be exposed to adversarial inputs, data poisoning, prompt manipulation, or compromised retrieval sources. Medical machine-learning systems have been shown to be susceptible to adversarial perturbations that can alter clinically relevant predictions while remaining difficult for humans to detect [99]. As more systems are built to support patient care by using electronic patient records as well as external knowledge and tools, the distinction between a model’s security and clinical safety will decrease.

The robustness of a model needs to be tested against specific failures that may occur in clinical scenarios. In addition to testing against random numbers, a model must be able to handle acquisition variability, missing input variables, distribution shifts, corrupted input, malicious use, and also failures of other services that the model interacts with. The security measures should be aligned with the least privilege principle in order to limit access to data and functions. Authenticated actions must be monitored and logged. Measures to recover from failures must also be implemented. Privacy and cybersecurity are components of clinical risk management and thus are not purely IT issues.

### 8.5. Human Oversight, Accountability, and Lifecycle Governance

Human oversight is effective only when responsibilities and intervention points are explicitly defined. A nominal human-in-the-loop configuration is insufficient when clinicians cannot reliably detect errors, lack sufficient time to interrogate outputs, or face excessive volumes of automatically generated recommendations. Formal regulatory oversight complements these governance principles. FDA frameworks for AI-enabled medical devices increasingly emphasize lifecycle management, transparency, post-deployment monitoring, and controlled modification, thereby shaping both system accountability and human oversight in clinical use [104]. The intensity of human oversight should be proportionate to clinical risk administrative tasks might only need to be audited after the fact, whereas diagnostic, therapeutic or even executable decisions need stronger prospective control points and a clear escalation procedure.

In addition to being governed before the AI system is deployed, the AI system must also be governed after it has been deployed. The behavior of a model can change without it being modified, due to changes in the patient population and clinical practice, as well as in the devices, coding systems, prevalence of diseases and in the data from upstream systems. Thus, post-deployment monitoring of the AI system tracks discrimination, calibration, performance in subgroups, missing values, override rates for human-in-the-loop for different clinical scenarios and outcomes of clinical relevance. The monitoring has predefined rules for investigation, for recalibration, for suspension of the model or for retraining of the model. The monitoring is part of the lifecycle of trustworthy AI, as described in the FUTURE-AI framework, and not just a step towards regulatory approval [23].

As AI systems become multimodal, generative, and increasingly agentic, this lifecycle approach becomes increasingly important. Governance is therefore the mechanism by which technical capability is converted into accountable clinical practice. Although this Review primarily focuses on clinician-facing AI, patient-facing transparency, informed consent, shared decision-making, and community accountability remain important considerations for responsible clinical implementation. The core dimensions of trustworthy clinical AI and their corresponding governance requirements are summarized in Table 6.

## 9. Persistent Challenges to Clinical Translation

The barriers that continue to limit the translation of artificial intelligence into routine healthcare are increasingly system-level rather than purely algorithmic. As clinical AI expands from task-specific prediction toward generative, multimodal, and agentic systems, successful deployment depends on the integrity of the underlying data, the reproducibility of the development process, compatibility with clinical information infrastructure, and the resources required to operate increasingly complex models. These constraints are particularly important because improvements in model capability do not inherently resolve weaknesses in the ecosystem in which the model is developed and deployed.

### 9.1. Data Quality, Reproducibility, and Shortcut Learning

Clinical datasets are rarely created specifically for machine learning. Electronic health records, medical images, physiological signals, and other routinely collected data reflect clinical workflows, acquisition devices, institutional practices, coding conventions, and patterns of missingness. Consequently, increasing dataset size does not necessarily increase dataset quality. Large datasets can preserve systematic artefacts and confounding structures that models may exploit more efficiently as their capacity increases.

Diagnostic datasets introduce additional sources of bias, including spectrum bias, partial or differential verification, incorporation bias when the reference standard includes model inputs, label leakage from downstream care, and imperfect or time-dependent reference standards. These biases can make a system appear diagnostically accurate without showing that it recognizes disease in the intended population. Cohort assembly, reference-standard adjudication, patient-level partitioning, and handling of indeterminate cases must therefore be reported and tested explicitly [7,8,9,105,106,107].

A particularly important manifestation is shortcut learning, in which a model achieves apparently high performance by exploiting features correlated with the target rather than clinically meaningful characteristics of the underlying disease. This phenomenon has been demonstrated across multiple forms of medical data. Ly et al. evaluated 13 clinical datasets encompassing 207,487 patients and five modalities, including radiographs, CT, ECG, clinical text, and auscultation data. Conventional evaluation overestimated external performance by approximately 20% on average because models learned hidden data-acquisition biases [105]. In radiography, models developed to detect pneumothorax have similarly exploited the presence of chest drains a clinically inappropriate shortcut because the device may indicate that the condition has already been diagnosed and treated [106].

Shortcut behaviour is not limited to obvious imaging artefacts. Acquisition pathways, institutional identifiers, demographic proxies, documentation patterns, preprocessing artefacts, and other latent characteristics can become predictive signals. Importantly, shortcut learning can coexist with excellent conventional test-set performance. Brown et al. demonstrated that shortcut testing can identify such behaviour in clinical machine-learning applications, reinforcing the need to evaluate what information a model is using rather than considering predictive accuracy alone [107].

Reproducibility is closely linked to this problem. Model architecture and performance metrics are insufficient to reconstruct an AI pipeline if cohort selection, patient-level partitioning, preprocessing, annotation, missing-data handling, feature construction, or hyperparameter selection are incompletely reported. Data leakage can further inflate performance when information from the evaluation set enters model development directly or indirectly. These concerns become harder to audit for foundation and proprietary models because the composition and provenance of pretraining datasets may be only partially disclosed. Reproducibility should therefore be considered a property of the complete analytical pipeline rather than of the trained model alone.

### 9.2. Interoperability and İnfrastructure

Clinical AI must also operate within a fragmented digital environment. Patient information is distributed across electronic health records, laboratory information systems, imaging archives, physiological monitoring platforms, genomic databases, and increasingly patient-generated data. Differences in terminology, in coding schemes, in user interfaces, in concepts for time and in architectures for health care data pose a significant barrier to the transfer of successful software solutions into clinical work flows.

To be clinically relevant for multimodal and agentic systems, such systems typically consist of several data streams, retrieval mechanisms, external knowledge resources, computational tools and model components. Interoperability for such systems goes far beyond simple data exchange and requires consistent semantics for example for patient and encounter identification, data provenance, version management, authentication and stable communication.

Interoperability is therefore a prerequisite for the effective integration of AI into clinical workflows. Isolated optimization of data pipelines or individual system components is insufficient to ensure safe and effective AI deployment in real-world clinical environments. Systems validated in small-scale studies must also demonstrate reliable integration with existing clinical data infrastructures and operational systems before routine deployment.

### 9.3. Computational and Environmental Sustainability

Increasing model complexity also introduces substantial computational and operational resource requirements. Large pre-training, multi-modal processing, repeated fine-tuning, large numbers of inferences, and agent-centric workflows all require significant amounts of hardware, memory, cloud-based computing resources, energy and maintenance resources to support development and deployment. The economic and environmental costs associated with these requirements may also affect clinical scalability and translation.

Environmental impact must not be confused with model size. A recent study on the use of autonomous AI for diabetic-eye disease screening found that the emissions attributable to AI-based image processing were substantially lower than those associated with conventional in-person screening pathways that required patient travel to hospital facilities. Under the assumptions of that study, the AI-enabled care pathway was associated with substantially lower estimated emissions when the broader screening pathway, including patient travel, was considered [108].

However, as AI deployment expands across health systems, the sum of many small computational costs can end up having large consequences for environmental sustainability. Therefore, whilst minimizing computation is an important goal, it is equally important to also maximize clinical value per computational resource. Model compression, efficient architectures, invoking large models only when required, deployment to local edge devices where appropriate, and the use of hybrid systems where large models are reserved for specific clinical use cases are key to enabling sustainable clinical deployment of AI within health systems.

In summary, scaling up clinical AI in the near term will require more than just increasing the size of the models. Data, reproducibility, interoperability, and computational resources must all be engineered to support the growth of AI in clinical care. Translation of AI to the clinic will increasingly depend on engineering an AI ecosystem rather than improving individual algorithms.

## 10. Emerging Frontiers

Future healthcare AI systems are unlikely to be dominated by a single model class. Instead, they will learn over time, operate over distributed data, maintain a patient specific computational representation, interact with the physical world and aid scientific discovery. The maturity of these emerging healthcare capabilities is heterogeneous with some, like federated learning, already being demonstrated in multicentre clinical studies while others like autonomous discovery and highly personalized digital twins are very much emerging health care AI capabilities and should be viewed with that status rather than as simply another clinical system awaiting deployment.

### 10.1. Adaptive and Distributed AI

Most clinical models are trained on a single fixed data set and then deployed as static systems. Continual learning enables models to adapt to newly available information while preserving performance on previously learned tasks. In medical imaging, continual learning approaches have been developed to exploit dynamic model memory across sequential tasks. Frameworks for standardized continual learning have also started to be developed for segmentation models that learn to adapt to changing data distributions over time [109,110]. The clinical rationale for continual learning is that disease prevalence, imaging distributions, hardware platforms, and clinical workflows evolve over time; models that can adapt safely to such changes may therefore offer advantages over systems requiring complete retraining. However, there is still the problem of control: a model’s adaptations should not unintentionally remove previously learned behavior from memory or introduce unknown behavior; therefore, update policies, model versions, and re-evaluation must all be explicitly controlled as opposed to simply allowing online learning.

Distributed AI addresses a different constraint: clinically useful data are often dispersed across institutions that cannot pool patient-level information. Federated learning enables collaborative model training while data remain locally held. Sheller et al. demonstrated multi-institutional medical modelling without centralizing patient data, and Dayan et al. subsequently trained a federated model across international institutions to predict clinical outcomes in patients with COVID-19 [111,112]. Edge-oriented implementations extend this logic by moving inference or learning closer to the data source, potentially reducing latency and dependence on centralized infrastructure [113]. These approaches do not eliminate privacy, heterogeneity, or governance problems, but they shift the architecture of clinical AI from centralized data aggregation toward coordinated computation across sites and devices.

### 10.2. Digital Twins and Personalized AI

Digital twins seek to construct computational representations that evolve with an individual patient and can be used to simulate, forecast, or compare possible future states. This is more demanding than conventional risk prediction because the representation is intended to remain dynamically linked to patient-specific information. Current healthcare studies illustrate several components of this vision rather than a fully realized general-purpose patient twin. ClinicalGAN, for example, used patient digital twins to support monitoring in clinical trials, while multiscale modelling of immune surveillance in micrometastases demonstrated how mechanistic simulation can contribute to cancer patient digital twins [114,115]. Other work has explored patient-specific treatment computation through in silico trials and graph-based forecasting of longitudinal medical conditions.

The principal opportunity is personalization at the level of trajectories and interventions rather than only static risk categories. A mature digital twin could potentially combine longitudinal observations, mechanistic knowledge, and learned representations to test alternative management strategies before they are applied to the patient. However, the evidence base remains fragmented across disease-specific models, simulations, and proof-of-concept systems. Personalized AI should therefore not be equated with the existence of a comprehensive virtual patient. Its clinical credibility will depend on whether the twin remains synchronized with changing patient states and whether simulated counterfactuals are sufficiently accurate to inform real decisions.

### 10.3. Embodied AI and Robotics

Embodied AI extends computational intelligence into systems that sense and act in the physical clinical environment. Surgical and medical robotics provide the clearest current pathway toward this form of AI. Research has progressed from teleoperation and constrained automation toward autonomous subtasks such as suturing, tissue manipulation, navigation, and instrument handling. Pedram et al. developed and quantified an autonomous suturing framework using a cable-driven surgical robot, while Wang et al. demonstrated a task-autonomous medical robot capable of incision stapling and staple removal [116,117]. Importantly, early human translation has also occurred: a first-in-men study evaluated a robotic tool providing autonomous inner-ear access for cochlear implantation [118].

Error potential of embodied AI systems differs from that of information systems as errors could cause immediate physical harm. The performance of embodied AI systems depends on perception, control, human anatomy, robotic embodiment, instrumentation, and robustness to unexpected events. In the near term, clinical deployment is likely to favour bounded autonomy, i.e., autonomous subtasks that have been validated for specific applications and that are used within larger clinician-supervised procedures. Autonomous tasks have to function fail-safe, have to be recoverable and must also provide evidence of their potential to increase precision and to support workflows without causing new procedural hazards.

### 10.4. Autonomous Scientific Discovery

A more nascent frontier is the use of AI to participate directly in the scientific process. Self-driving laboratories combine machine learning with automated experimentation so that hypotheses or candidate solutions can be generated, tested, and iteratively refined with reduced manual intervention. Rapp et al. demonstrated an autonomous laboratory that navigated protein fitness landscapes by integrating machine-learning-guided selection with experimental measurement [119]. This shifts AI from analysing completed experiments toward selecting which experiment should occur next.

The frontier is now beginning to intersect more directly with biomedical discovery. A 2026 study reported an agentic framework for autonomous scientific discovery in cancer pathology, indicating that coordinated AI systems can move beyond isolated prediction toward multi-step scientific analysis [120]. To connect literature, computational studies, hypothesis generation, experimental design and the control of robots in closed or semi-closed loops by means of AI to generate reproducible biological knowledge, novel drugs or testable hypotheses of clinical relevance in medicine using research approaches more efficiently than currently practiced requires a body of evidence currently small in size and automation level.

Most research in AI today is moving across several fronts, and in our view the biggest transition happening currently is from fixed, centralized and for the most part task-specific AI systems, to more adaptive, distributed, patient-specific, physically interactive and even scientifically generative systems. While there is variable evidence of the maturity of these new systems, notable progress has been made recently in the areas of federated learning and bounded robotic autonomy, but evidence of the adequacy of current evidence for the bulk of more complex digital twins and fully autonomous scientific discovery remains largely at the level of proof-of-concept.

## 11. Conclusions

Artificial intelligence is expanding across the diagnostic continuum, from screening and triage to disease detection, differential diagnosis, prognostic stratification, and treatment-response assessment. Predictive models remain central to estimating classes and risks; generative models can synthesize histories and support differential reasoning; multimodal systems can integrate images, signals, text, pathology, and molecular data; and agentic architectures can coordinate evidence retrieval, tools, and sequential workflow steps. These paradigms are increasingly convergent, but technological convergence does not itself establish diagnostic value.

The diagnostic question must remain the organizing principle. Every system should specify its intended population, target condition and alternatives, role in the pathway, reference standard, operating threshold, and consequences of false-positive, false-negative, and indeterminate results. High retrospective accuracy, fluent generation, multimodal fusion, or autonomous task completion is insufficient if performance is poorly calibrated, fails under spectrum or prevalence shift, omits critical alternatives, or does not change an appropriate clinical decision. A credible implementation pathway therefore progresses from internal performance to independent external and prospective validation, human–AI and workflow evaluation, comparative clinical-impact studies, and post-deployment surveillance. Outcomes should include diagnostic yield, time to diagnosis, additional testing, downstream harm, equity, resource use, and patient-relevant benefit.

Trustworthiness is consequently a property of the complete diagnostic system rather than a checklist of model attributes. Fairness, transparency, privacy, security, accountability, and robustness must be engineered across the data, model, interface, users, institution, and monitoring process. In generative and agentic systems, provenance, uncertainty, bounded permissions, audit trails, abstention, error recovery, and escalation to a clinician are essential because errors can propagate across multiple steps.

Emerging approaches, including continual and federated learning, patient digital twins, embodied intelligence, and autonomous scientific discovery, may extend this ecosystem, but their diagnostic relevance must be demonstrated rather than inferred from novelty. The next generation of healthcare AI should be judged by whether rigorously evaluated human–AI systems improve disease detection, differential diagnosis, prognostic stratification, and care decisions safely, equitably, and reproducibly in real clinical settings. Clinical translation is achieved not when a model produces an impressive output, but when its use produces measurable and durable improvements in the diagnostic pathway, clinically relevant care processes, or patient-relevant outcomes.

## Figures and Tables

**Table 1:** Clinical and translational profile of major AI paradigms in healthcare.

AI Paradigm	Key Diagnostic Uses	Principal Benefit	Key Hazard	Highest-Risk Failure	Current Evidence Level	Evaluation Priority
**Predictive AI**	Detection, classification, risk and prognostic stratification	Consistent estimation of predefined clinical endpoints	Poor calibration and limited transportability	False reassurance or inappropriate intervention under distribution shift	Most mature; external and prospective evidence available in selected applications	External/prospective validation, calibration, clinical impact
**Generative AI**	Differential diagnosis, synthesis of clinical information, documentation and decision support	Contextual synthesis and communication of complex information	Hallucination, omission, and automation bias	Plausible but incorrect output influencing clinical reasoning	Predominantly benchmark and retrospective; prospective/workflow evidence remains limited	Factual reliability, uncertainty, abstention/escalation, human–AI evaluation
**Multimodal AI**	Integration of imaging, text, EHR, signals, pathology, and molecular data	Integration of complementary evidence across modalities	Modality imbalance, missing data, and integration failure	Undetected propagation of missing or discordant information	Mainly retrospective; external validation emerging	Incremental value over unimodal models, missing-modality robustness, external/prospective validation
**Agentic AI**	Multi-step reasoning, evidence retrieval, tool use, workflow coordination	Execution and coordination of complex clinical tasks	Error propagation and unsafe tool/action execution	Incorrect autonomous action affecting downstream care	Early; largely proof-of-concept and task-level evidence	End-to-end task safety, tool failures, recovery, escalation, human override

**Table 2:** Evidence domains for the clinical translation of diagnostic and predictive artificial intelligence.

Evaluation Domain	Core Question	Typical Study Design	Key Outcomes	Interpretation	Supporting Framework
Internal performance	Does the model perform reliably within the development setting?	Bootstrap, cross-validation, held-out test set	Discrimination, calibration, prediction error	Establishes predictive performance; does not demonstrate transportability	TRIPOD+AI [9]
External validation	Does performance persist in independent data?	Temporal, geographic, cross-institutional, or cross-population validation	Discrimination, calibration, subgroup performance	Tests transportability beyond model development	TRIPOD+AI [9]; Cabitza et al. [8]
Prospective evaluation	Does performance persist when applied to consecutively encountered patients?	Prospective cohort or multicentre evaluation	Prospective discrimination, calibration, failure rate	Tests robustness under contemporary clinical conditions	DECIDE-AI [10]
Workflow and human–AI evaluation	Does AI alter decisions or clinical processes in a useful and safe manner?	Silent deployment, clinician–AI study, implementation study	Decision changes, time, workload, alert burden, human factors	Establishes operational usefulness and interaction with users	DECIDE-AI [10]
Clinical-impact evaluation	Does AI-assisted care improve clinically meaningful processes or patient outcomes?	Controlled, pragmatic, cluster-randomised, or randomised trial	Patient outcomes, adverse events, resource use, clinically relevant process outcomes	Provides the strongest evidence of clinical utility	CONSORT-AI [21]; SPIRIT-AI [22]
Post-deployment surveillance	Are performance and safety maintained after implementation?	Real-world monitoring, registry, continuous surveillance	Dataset/model drift, calibration, subgroup performance, safety events	Assesses durability, equity, and emerging harms	FUTURE-AI [23]

**Note.** These domains are not necessarily sequential or mutually exclusive; external, prospective, workflow, and impact evaluations may overlap within the same study. The framework synthesises principles from TRIPOD+AI, DECIDE-AI, CONSORT-AI, SPIRIT-AI, FUTURE-AI, and methodological work on external validation [8,9,10,21,22,23].

**Table 3:** Representative primary studies illustrating the clinical value and limitations of multimodal AI.

Study	Clinical Task	Modalities	Key Contribution	Translational Limitation
Boehm et al., 2022 [62]	Ovarian cancer risk stratification	CT + histopathology + genomics + clinical data	Complementary multimodal features improved risk stratification.	Single disease setting; broader validation required.
Qiu et al., 2022 [63]	Dementia assessment	Clinical data + neuropsychology + neuroimaging + functional measures	Flexible combinations supported multi-step dementia assessment.	Availability and composition of modalities vary across settings.
Khader et al., 2023 [65]	Multi-label chest diagnosis	Chest radiographs + clinical parameters	Transformer-based integration outperformed single-modality models for several tasks.	Retrospective evaluation; gain is task-dependent.
Zhou et al., 2024 [59]	Dermatological diagnosis	Skin images + clinical concepts/text	Demonstrated interactive multimodal diagnostic capability on real cases.	Specialty-specific evaluation; deployment safety remains unresolved.
Tanno et al., 2025 [60]	Radiology report generation	Chest radiographs + language	Demonstrated clinician-VLM collaboration and expert evaluation of generated reports.	Clinically significant errors persisted.
Huang et al., 2020 [68]	Pulmonary embolism detection	CT pulmonary angiography + EHR	Late fusion outperformed imaging-only and EHR-only models.	Single use case; workflow benefit was not directly tested.
Vale-Silva & Rohr, 2021 [69]	Pan-cancer survival	Clinical + imaging + multi-omics	Integrated heterogeneous modalities and accommodated missing modalities.	Retrospective datasets; clinical utility requires prospective testing.
Liu et al., 2023 [70]	Clinical prediction with incomplete data	Structured + unstructured clinical modalities	Attention/contrastive fusion explicitly addressed missing modalities.	Robustness depends on missingness patterns represented during development.

Abbreviations: CT = computed tomography; EHR = electronic health record; VLM = vision–language model.

**Table 4:** Representative primary studies of agentic AI in healthcare.

Study	Clinical Task	Agentic Properties	Evaluation Focus	Autonomy Level	Key Limitation
Autonomous oncology agent [72]	Oncology decision-making	Autonomous clinical reasoning and decision support	Development and validation	Bounded autonomous	Bounded task; broader prospective deployment needed.
Custom LLM agents [73]	Evidence-based medicine	Retrieval/tool-augmented evidence synthesis	Accuracy versus non-custom models	Human-supervised	Task performance does not establish workflow safety.
Radiotherapy planning agents [74]	Radiotherapy planning	Sequential tool use and planning	Feasibility of automated planning	Human-supervised	High-consequence actions require stringent oversight.
LLM multi-agent order sets [75]	Clinical order-set optimisation	Multi-agent critique and optimisation	Quality of generated order sets	Human-supervised	Downstream patient outcomes not established.
Neuro-ophthalmic MAS [76]	Diagnosis and treatment planning	Role-specialised multi-agent reasoning	Diagnostic and planning performance	Human-supervised	Specialty-specific evaluation.
CARE-AD [77].	Alzheimer’s disease prediction	Multi-agent reasoning over longitudinal notes	Predictive performance	Human-supervised	Retrospective clinical-note setting.
Safety-aware multi-agent screening [78]	NSCLC trial pre-screening	Rule-based vs single- vs multi-agent orchestration	Safety and comparative performance	Human-supervised	Proof-of-concept evidence.
Clinical identity failures [80]	Patient-specific clinical tasks	Agent interaction with patient context	Silent identity-error failure	Bounded autonomous	Demonstrates system-level rather than answer-level risk.

**Table 5:** Major data modalities used by artificial intelligence across clinical specialties.

Clinical Specialty	Major Data Modalities Used in AI	Representative Data
**Radiology and medical imaging**	Medical imaging; clinical/EHR data; text	CT, MRI, radiography, mammography, ultrasound, PET, radiology reports
**Pathology**	Digital pathology; imaging; molecular/omics; clinical data	Whole-slide images, histology, cytology, genomic and molecular profiles
**Cardiology**	ECG; imaging; physiological monitoring; EHR; wearables	12-lead ECG, Holter ECG, echocardiography, cardiac CT/MRI, PPG, blood pressure
**Neurology**	EEG/EMG; neuroimaging; EHR; wearables; omics	EEG, EMG, evoked potentials, MRI, CT, PET, gait and movement sensors
**Oncology**	Imaging; digital pathology; omics; EHR; laboratory data	CT/MRI/PET, histology, genomics, transcriptomics, biomarkers, treatment records
**Psychiatry and mental health**	Clinical text; EHR; EEG; audio; wearables	Clinical notes, questionnaires, EEG, speech, sleep, activity and smartphone-derived signals
**Gastroenterology**	Endoscopic video/images; pathology; EHR; laboratory data	Colonoscopy, gastroscopy, capsule endoscopy, histology, biochemical markers
**Ophthalmology**	Ocular imaging; clinical data	Fundus photography, OCT, OCTA, slit-lamp and retinal imaging
**Dermatology**	Clinical/dermoscopic imaging; pathology; clinical data	Smartphone photographs, dermoscopy, histopathology
**Physical medicine and rehabilitation**	Wearables/sensors; video; EMG; clinical outcomes	Accelerometry, inertial sensors, gait, motion capture, surface EMG, functional scores
**Orthopaedics**	Imaging; sensor/biomechanical data; EHR	Radiography, CT, MRI, gait, force and motion measurements
**Emergency and critical care**	EHR; laboratory data; ECG; continuous physiological signals	Vital signs, ECG, SpO2, blood pressure, laboratory trajectories, medication and intervention data
**Surgery and perioperative medicine**	Video; imaging; physiological monitoring; EHR	Operative video, endoscopy, intraoperative imaging, ECG, blood pressure, SpO2
**Pulmonology**	Imaging; physiological signals; audio; EHR	Chest CT/X-ray, spirometry, SpO2, respiratory waveforms, cough and breath sounds
**Nephrology**	EHR; laboratory data; pathology; imaging; omics	Creatinine/eGFR trajectories, urine biomarkers, renal histology, ultrasound, molecular profiles
**Obstetrics and gynecology**	Ultrasound/imaging; physiological signals; EHR; laboratory/omics	Fetal ultrasound, cardiotocography, maternal records, laboratory and genomic data
**Pediatrics and neonatology**	EHR; physiological monitoring; EEG; imaging; audio/video	Neonatal EEG, vital signs, imaging, cough/cry audio, developmental video
**Infectious diseases**	EHR; laboratory/microbiology; physiological monitoring; text/omics	Microbiology, antimicrobial susceptibility, vital signs, laboratory trends, genomic sequencing

**Table 6:** Core dimensions of trustworthy clinical AI and evidence required for governance.

Dimension	Principal Risk	Evidence Required	Governance Response	Representative Refs
Fairness and equity	Unequal error rates; proxy discrimination	Subgroup/intersectional discrimination, calibration, error severity	Predefined fairness analysis; mitigation; equity monitoring	[95,96,102]
Transparency and explainability	Plausible but unfaithful explanations; inappropriate reliance	Provenance, uncertainty, explanation fidelity, human–AI interaction	Intended-use documentation; auditability; user training	[23,97,98]
Privacy and security	Data leakage, adversarial manipulation, compromised inputs	Threat modelling, privacy testing, adversarial/robustness evaluation	Least privilege; access control; logging; incident response	[23,99]
Human oversight	Automation bias; unclear responsibility	Override behaviour, intervention effectiveness, failure recognition	Risk-proportionate human review; escalation; accountability	[23,97]
Lifecycle governance	Dataset shift, model drift, changing workflow	Prospective performance, calibration, subgroup and outcome monitoring	Monitoring thresholds; recalibration, suspension or retraining	[23,100,101]

## Data Availability

No new data were created or analyzed in this study. Data sharing is not applicable to this article.

## Supplementary Materials

The following supporting information can be downloaded at: https://www.mdpi.com/article/10.3390/bioengineering13101175/s1, Table S1. Representative diagnostic and clinically translational applications of artificial intelligence across healthcare [121,122,123,124,125,126,127,128,129,130,131,132,133,134,135,136,137,138,139,140].

## Abbreviations

AI	Artificial intelligence
ML	Machine learning
DL	Deep learning
LLM	Large language model
EHR	Electronic health record
NLP	Natural language processing
VLM	Vision–language model
RAG	Retrieval-augmented generation
XAI	Explainable artificial intelligence
CAD	Computer-aided diagnosis
CT	Computed tomography
MRI	Magnetic resonance imaging
ECG	Electrocardiography
EEG	Electroencephalography
IoT	Internet of Things
FL	Federated learning
