# Supplementary material for: Artificial Intelligence in Healthcare: From Predictive Models to Generative, Multimodal, and Agentic AI
Source: Bioengineering (Basel). 2026 Oct 8;13(10):1175. doi: 10.3390/bioengineering13101175 (PMC13701811; doi:10.3390/bioengineering13101175)
Supplement: Supplementary file 1 [file bioengineering-13-01175-s001.zip › bioengineering-4580959-supplementary.pdf]

Table S1. Representative diagnostic and clinically translational applications of artificial intelligence across healthcare

Clinical specialty	Representative study	AI paradigm	AI task	Data modality	Clinical application	Clinical evidence	Key clinical finding
**Radiology & medical imaging**	Lång et al., 2023 [81]	Deep learning / computer vision	Detection / screening	Mammography	AI-supported mammography screening	Randomized controlled screening study	AI-supported screen reading was prospectively evaluated against standard double reading in population screening.
**Radiology & medical imaging**	Saha et al., 2024 [88]	Deep learning / computer vision	Detection / classification	Multiparametric MRI	Prostate cancer detection	International paired non-inferiority study	AI was compared with radiologists for clinically significant prostate cancer detection on MRI.
**Pathology**	Pantanowitz et al., 2020 [41]	Deep learning / computer vision	Classification / detection	Whole-slide histopathology	Prostate cancer diagnosis	Blinded clinical validation and deployment study	The algorithm was clinically validated and deployed for prostate core-biopsy whole-slide interpretation.
**Pathology**	Hollon et al., 2020 [86]	Deep learning	Classification	Stimulated Raman histology	Intraoperative brain-tumour diagnosis	Prospective clinical validation	Deep learning enabled near-real-time intraoperative diagnostic classification from label-free histology.
**Cardiology**	Yao et al., 2021 [121]	Deep learning	Classification / screening	12-lead ECG	Detection of reduced ejection fraction	Pragmatic cluster-randomized clinical trial	AI-enabled ECG increased identification of patients with low ejection fraction in routine practice.
**Cardiology**	Noseworthy et al., 2022 [122]	Deep learning	Risk stratification / screening	12-lead ECG	Atrial-fibrillation screening	Prospective non-randomized interventional trial	AI-guided ECG screening enriched identification of patients suitable for atrial-fibrillation monitoring.
**Neurology**	Xue et al., 2024 [87]	Multimodal AI / machine learning	Differential diagnosis / prediction	Multimodal clinical data	Differential diagnosis of dementia etiologies	Multicohort clinical validation	Multimodal AI was evaluated for differentiating dementia etiologies across heterogeneous patient information.
**Neurology**	Shi et al., 2020 [89]	Deep learning / computer vision	Detection	CT angiography	Intracranial aneurysm detection	Multicentre clinical validation	The model supported clinically applicable aneurysm detection on CT angiography.
**Oncology**	Klein et al., 2021 [123]	Machine learning	Classification / screening	Cell-free DNA methylation	Multi-cancer early detection	Independent clinical validation	A targeted methylation-based classifier was validated for multicancer detection and cancer signal origin prediction.
**Oncology**	Feng et al., 2022 [124]	Multimodal AI / radiopathomics	Prediction	MRI + pathology/clinical features	Neoadjuvant treatment-response prediction	Multicentre observational validation study	Radiopathomic integration predicted pathological complete response in locally advanced rectal cancer.

**Psychiatry & mental health**	Fulmer et al., 2018 [125]	Conversational AI	Intervention	Text-based conversational data	Depression and anxiety symptom support	Randomized controlled trial	A conversational AI intervention was evaluated for reducing symptoms of depression and anxiety.
**Psychiatry & mental health**	Webb et al., 2019 [126]	Machine learning	Prediction / treatment-response stratification	Clinical and trial data	Antidepressant-response prediction	Secondary analysis of a clinical trial cohort	Machine learning was used to personalize prediction of antidepressant versus placebo response.
**Gastroenterology**	Wang et al., 2019 [84]	Deep learning / computer vision	Real-time detection	Colonoscopy video	Polyp and adenoma detection	Prospective randomized controlled trial	Real-time AI assistance increased polyp and adenoma detection during colonoscopy.
**Gastroenterology**	Repici et al., 2020 [85]	Deep learning / computer vision	Real-time detection	Colonoscopy video	Colorectal neoplasia detection	Multicentre randomized controlled trial	Computer-aided detection increased adenoma detection during routine colonoscopy.
**Ophthalmology**	Ipp et al., 2021 [82]	Deep learning / autonomous AI	Classification / screening	Fundus photography	Diabetic-retinopathy screening	Prospective pivotal clinical evaluation	Autonomous AI was evaluated for detection of referable and vision-threatening diabetic retinopathy.
**Ophthalmology**	Ruamviboonsuk et al., 2022 [83]	Deep learning	Classification / screening	Retinal fundus images	Diabetic-retinopathy screening	Prospective interventional multisite cohort	Real-time deep-learning screening was evaluated within a national multisite programme.
**Dermatology**	Marchetti et al., 2023 [90]	Deep learning / computer vision	Classification	Dermoscopy	Melanoma diagnosis	Prospective clinical validation	Open-source dermoscopy AI underwent prospective validation for melanoma diagnosis.
**Dermatology**	Menzies et al., 2023 [91]	Deep learning / mobile AI	Classification / decision support	Clinical and dermoscopic images	Pigmented skin-cancer diagnosis and management	Multicentre prospective diagnostic trial	Mobile AI was prospectively compared with human assessment for diagnosis and management decisions.
**Physical medicine & rehabilitation**	Jayakumar et al., 2021 [127]	AI-enabled decision support	Decision support	Patient-reported and clinical data	Knee-osteoarthritis shared decision-making	Randomized clinical trial	An AI-enabled decision aid was evaluated for decision quality, shared decision-making, experience, and functional outcomes.
**Physical medicine & rehabilitation**	Marcuzzi et al., 2023 [128]	AI-enabled digital intervention	Personalization / intervention	Patient-reported digital data	Neck and low-back pain self-management	Randomized clinical trial	An AI-based self-management application was tested for musculoskeletal health outcomes.
**Orthopaedics**	Auloge et al., 2020 [129]	AI-assisted navigation / augmented reality	Navigation / procedural guidance	Procedural imaging	Percutaneous vertebroplasty guidance	Pilot randomized clinical trial	AI/AR-based navigation was prospectively evaluated during vertebroplasty.

**Orthopaedics**	Duron et al., 2021 [92]	Deep learning / computer vision	Detection	Radiography	Appendicular skeletal fracture detection	Multicentre diagnostic study	AI assistance was assessed for fracture detection by emergency physicians and radiologists.
**Emergency & critical care**	Adams et al., 2022 [130]	Machine learning	Prediction / early warning	EHR + vital signs + laboratory data	Sepsis early warning	Prospective multisite implementation study	TREWS was evaluated after real-world deployment for its association with sepsis care and patient outcomes.
**Emergency & critical care**	Levin et al., 2018 [131]	Machine learning	Risk stratification / triage	EHR triage data	Emergency-department triage	Clinical validation study	Machine-learning electronic triage improved differentiation of patients by subsequent clinical outcomes versus ESI.
**Surgery & perioperative medicine**	Wijnberge et al., 2020[20]	Machine learning	Prediction / early warning	Continuous arterial-pressure waveform	Intraoperative hypotension prevention	Randomized clinical trial	A machine-learning early-warning system reduced the depth and duration of intraoperative hypotension.
**Surgery & perioperative medicine**	Brennan et al., 2019 [132]	Machine learning / decision support	Risk prediction	Preoperative EHR/clinical data	Postoperative complication risk assessment	Pilot usability study	MySurgeryRisk was compared with clinician judgment as a preoperative risk-assessment aid.
**Pulmonology**	Ardila et al., 2019 [133]	Deep learning / computer vision	Detection / risk prediction	Low-dose chest CT	Lung-cancer screening	Retrospective development with external validation	Three-dimensional deep learning was externally validated for lung-cancer risk estimation from screening CT.
**Pulmonology**	Sinha et al., 2020[134]	Machine learning	Phenotyping / classification	Clinical and laboratory data	ARDS phenotype classification	Secondary analysis of randomized trials	Parsimonious algorithms reproduced clinically relevant ARDS phenotypes using routinely available variables.
**Nephrology**	Chen et al., 2019 [135]	Machine learning / statistical prediction	Risk prediction / stratification	Clinical + laboratory data	Kidney-outcome prediction in IgA nephropathy	Clinical cohort validation	A risk model stratified patients according to subsequent kidney outcomes.
**Nephrology**	Kers et al., 2022 [136]	Deep learning / computer vision	Classification	Kidney-transplant histopathology	Transplant pathology classification	Retrospective multicentre proof-of-concept study	Deep learning classified kidney-transplant pathology across multiple centres.
**Obstetrics & gynecology**	Illingworth et al., 2024[137]	Deep learning / computer vision	Ranking / selection	Embryo images	Embryo selection in IVF	Randomized double-blind non-inferiority trial	Deep-learning embryo selection was prospectively compared with manual morphology-based selection.
**Obstetrics & gynecology**	Artzi et al., 2020 [138]	Machine learning	Risk prediction	Nationwide EHR	Gestational-diabetes prediction	Nationwide retrospective	EHR-based machine learning identified women at risk of gestational diabetes.

						cohort with validation	
**Pediatrics**	Pavel et al., 2020 [139]	Machine learning	Detection / classification	Neonatal EEG	Neonatal seizure recognition	Multicentre randomized controlled trial	Machine-learning seizure recognition was evaluated for its effect on neonatal seizure identification.
**Pediatrics**	Porter et al., 2019 [93]	Machine learning / audio analytics	Classification	Cough audio	Paediatric respiratory diagnosis	Prospective multicentre diagnostic study	Automated cough-sound analysis was prospectively evaluated for common respiratory disorders in children.
**Infectious diseases**	Mao et al., 2018[94]	Machine learning	Prediction / early warning	Vital-sign time series	Sepsis prediction	Multicentre validation study	A vital-sign-based algorithm was validated across emergency, ward, and ICU settings.
**Infectious diseases**	Giannini et al., 2019 [140]	Machine learning	Prediction / early warning	EHR + clinical data	Severe sepsis and septic-shock prediction	Development, implementation, and clinical-impact study	The model progressed from development to implementation and evaluation of its effect on clinical practice.
